# Asking a question

**DOI:** 10.1128/jb.00050-24

**Published:** 2024-05-31

**Authors:** Roberto Kolter

**Affiliations:** 1 Department of Microbiology, Harvard Medical School, Boston, Massachusetts, USA; Geisel School of Medicine at Dartmouth, Hanover, New Hampshire, USA

**Keywords:** science, philosophy

## Abstract

While scientific research should be carried out objectively, the choices of questions asked and approaches taken are deeply personal and subjective. I urge individuals to pursue questions they love and to periodically scrutinize the reasons (the philosophies) that drive that love. As a case study, I scrutinize the “whys” behind some of the scientific questions I pursued during my career.

## COMMENTARY

The initial idea of this essay dates back to several years ago, perhaps more than I care to admit. In his role as co-director, George O'Toole asked me to give a lecture in the Woods Hole Microbial Diversity summer course. His suggested title for my lecture was the same as this essay’s: “Asking a Question.” What did he mean by that? In his words: “*...it might be fun to have you give a lecture on the theme of 'asking a question,' to talk about some projects from your lab over the years in the context of formulating a scientific question.*” I took quite a few liberties interpreting these words and decided to examine the personal philosophies that guide our choice of the scientific questions we decide to ask. In discussions we had after I had given the lecture, George suggested that it might also be fun for me to compose a short essay on the same topic. I agreed and, because of my tendency to procrastinate in the absence of a strict deadline, I have had a lot of time to think about “asking a question.” Here, finally, I share with you some of those thoughts.

I will begin by asking you, the reader, to answer this: What are your favorite works of art? Stop reading, close your eyes, and for a few moments, ponder this question.

Whether you recalled paintings, works of music, or literature, of one thing I can be certain. The selections that come to mind among all those reading this will be strikingly diverse. You will each have your reasons as to why you made your choices, and these reasons will be distinctly personal. The point of this brief mental exercise might already be apparent. In pursuing a career in science, you will no doubt be asking questions and seeking answers. That is what science is. We ask questions about the world around us, and then we proceed to find a path toward answering them. That you already know. But the point I want to make is that, in my opinion, it is key to understand the why and how you decide to ask a question. Why do I consider this introspection so important? As we seek and explore questions, the paths we follow should be consonant with our personal worldview. If we enter an area of inquiry with closed eyes, more swayed by others’ opinions than our own, we might one day open our eyes only to discover we are lost.

For anyone beginning to think about asking a scientific question, I recommend paying close attention to the following. First and foremost, recognize that the selection of what question you ask is deeply personal; make sure you scrutinize the reasons behind why you are asking that question. I believe you should truly love the questions you are asking, and thus it behooves you to ponder upon the reasons why you love that question. These being very much personal reflections, the answers that arise should be in keeping with your personal philosophy. Undoubtedly, these answers will be different for each individual and, while the scientific method aims at objectivity, the choice of topic of investigation and the approaches to be taken will without doubt be subjective. Whatever your reasons are, make sure you examine them and do not go pursuing questions blindly.

Since I have argued that the process of arriving at a question is a personal one, I now will describe what I see, in retrospect, as the “whys” behind some of the questions I asked along my career with the hope of conveying what it was that I loved about them and how these reasons evolved alongside my personal philosophy.

**Fig 1:**
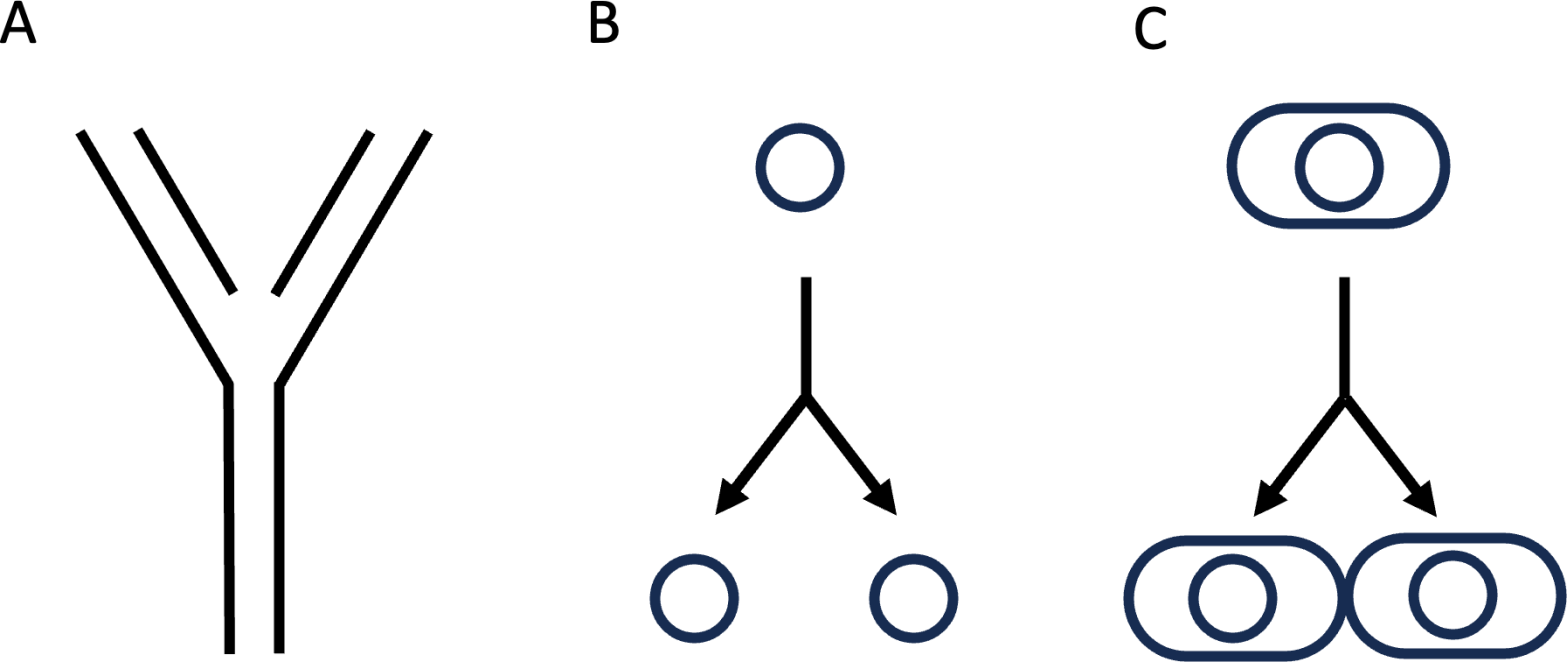
DNA (**A**) complementary strands provide the mechanism for faithful replication, (**B**) one DNA circle becomes two, and (**C**) DNA replication within the cell.

As investigators interested in the life sciences, I think it is undeniable that at the heart of the questions we ask is the BIG question: What is life? While that is the underlying big question, it is one that can be approached from myriad different angles; let us call these angles smaller “sub-questions.” So what particular sub-question resonated with me early on in my career, in the 1970s? There is no doubt that, like for many others of my generation, the intrinsic beauty of the DNA double helix (whose discovery coincided with the year of my birth) had a captivating effect. Personally, this beauty became even greater by the simple explanatory message of one of its key features, the complementary strands provided the mechanism for faithful replication (Fig. 1A). I felt that somehow, therein, were many of the answers to the question “what is life?” Understanding the double helix function would lead to understanding life. What I loved was the simplicity of replication. Simplifying it in my mind even further, the question became how does one circle become two? (Fig. 1B) But what really attracted me was how this replication could occur inside the cell such that each daughter cell inherited a copy of the DNA (Fig. 1C). How is that process regulated? How is the information for life maintained?

Why choose to study regulation? I had been profoundly influenced by Jacob and Monod (1) and others who worked out the mechanisms of the regulation of gene expression. Thus, when Jacob, Brenner, and Cuzin proposed that DNA replication could be regulated in an analogous manner to operons in stating their “replicon hypothesis” (2), I was smitten. Their question was very easily framed: how are replicons regulated? Part of the beauty of this question was its simplicity. From it came a simple and testable hypothesis. A replicon encodes a protein that recognizes a site on the DNA (the origin of replication) and acts positively to enable replication. To address this question, what was needed was a simplified (small) replicon that could be easily dissected. Recombinant DNA methodologies that emerged in the 1970s made this possible.

**Fig 2:**
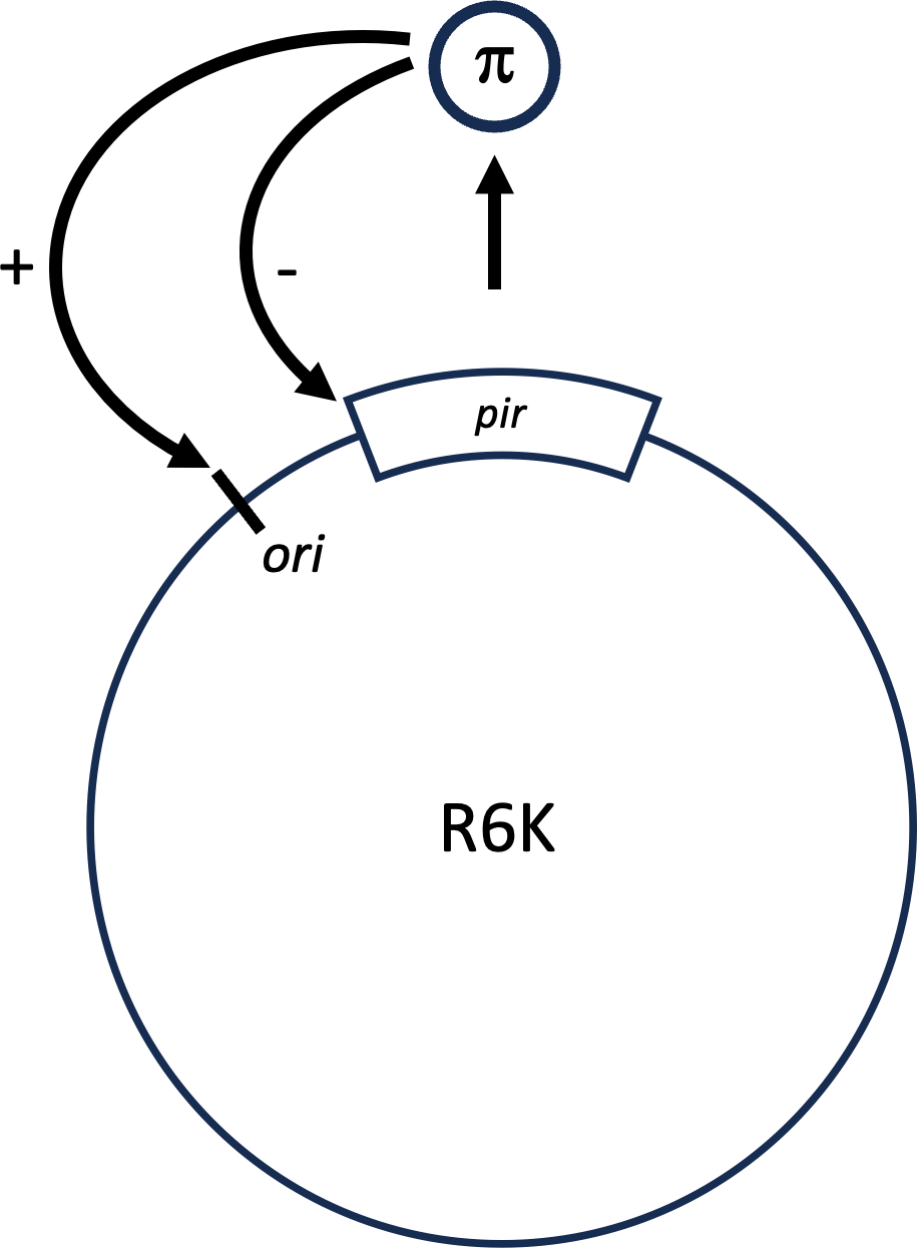
Model for the regulation of replication of plasmid R6K; adapted from reference (3).

For nearly 4 years, I focused on this question studying the replication of plasmid R6K in Donald Helinski’s lab. Our efforts paid off nicely, and by the end of my Ph.D. training, we had found that regulation of replication was achieved by an initiator protein (π) that bound to a specific sequence in the DNA, the origin. In addition, the protein bound to the promoter of its own gene (*pir*) to regulate its concentration inside the cell (Fig. 2) (3). That was it.

**Fig 3:**
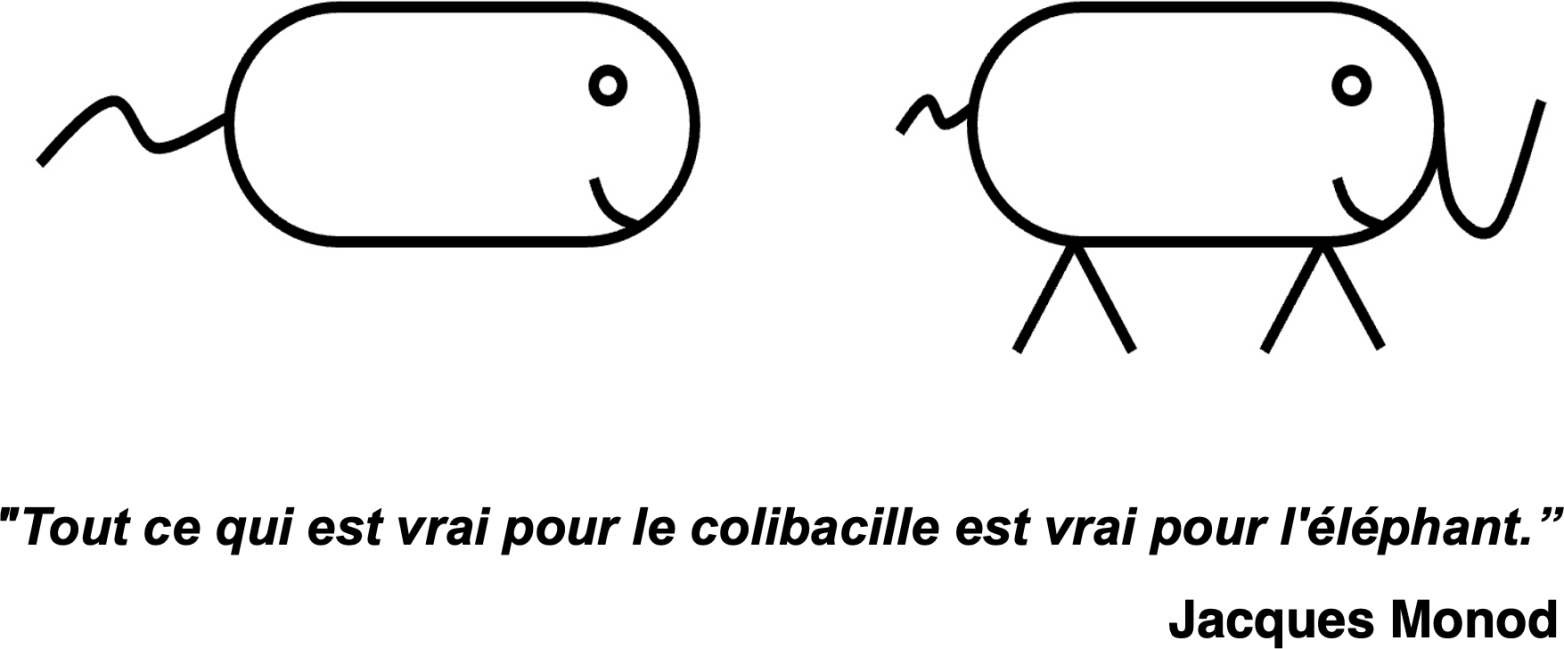
All that is true for *E. coli* is true for the elephant.

To me, the question was answered. Done. Yet, to others, this was only the beginning. And that is the important message here. Neither I nor the others were right. It was simply what each one of us loved. I had liked the simplicity of our answers. Other saw in those results the beginning of unraveling the complexity. I was, and to some degree continue to be, moved by questions that can yield clear, simple answers. Perhaps that is why I felt enamored by the elegant reductionism of Jacob and Monod, epitomized in the phrase “*What is true for E. coli is true for the Elephant*” (Fig. 3) (1). Much later I would learn, from Dianne Newman, that the phrase likely was derived from Albert Jan Kluyver who in the 1920s had stated “from elephant to butyric acid bacterium - it’s all the same” to drive home the universality of the biochemistry of central metabolism (4). The beauty was that, like metabolism, gene function was proving to have some universal aspects as well.

But what was really behind my love for understanding how DNA molecules replicate? For that, I need to go back to 10 years before the discovery of the double helix. Again, to the question, *what is life*? My interest in molecular biology and its history inevitably had me reading Erwin Schrödinger’s influential essay “*"What is Life*?” (5). Plodding through his explanations of Delbrück’s prior speculations on the nature of the gene was not an easy task. But the experience proved instructive. Yet, what I was most taken by was the epilogue of Schrödinger’s essay, enticingly entitled “*On Determinism and Free Will.*” As a quantum physicist interested in understanding life, he found himself seeking to reconcile two initially contradictory premises. On the one hand, there is the physicist’s conclusion that, from genes to the whole organism, “*my body functions as a pure mechanism according to the Laws of Nature”* (determinism). On the other hand, regarding that very same body, he writes: “*I know, by incontrovertible direct experience, that I am directing its motions*” (free will). In attempting to reconcile these apparently contradictory premises, he concludes:

“The only possible inference from these two facts is, I think, that I – I in the widest meaning of the word, that is to say every conscious mind that has ever said or felt 'I' – am the person, if any, who controls the 'motion of the atoms' according to the Laws of Nature... ... it is daring to give to this conclusion the simple wording that it requires. In Christian terminology to say: 'Hence I am God Almighty' sounds both blasphemous and lunatic.” (5)

No reconciling the premises there. Instead, Schrödinger found reconciliation in the ancient writings of Advaita Vedanta philosophy. For the uninitiated and brief to the extreme, this branch of Hindu philosophy, based largely on the Bhavagad Gita (6) and the Upanishads (7), interprets these writings in a non-dualistic way, as Schröndinger states:

“From the early Upanishads the recognition ATHMAN = BRAHMAN (the personal self equals the omnipresent, all-comprehending eternal self) was in Indian thought considered, far from being blasphemous, to represent the quintessence of deepest insight into the happenings of the world.” (5)

Having myself been steeped in English translations of the Ten Principal Upanishads (7) and the Bhagavad Gita (6), I found great satisfaction in learning that such an influential physicist as Schrödinger framed his scientific knowledge, from quantum mechanics to molecular genetics, in this non-dualistic philosophical worldview: ATMAN = BRAHMAN. I came to realize that my efforts to understand, first DNA replication and then the mechanisms of life and death in the stationary phase (8), were ways to inquire not only about “What is life?” but also about “Who am I?” Here is an example. I could see the universality of biochemistry and gene function contained within the broad reaches of this remarkably insightful verse from the Isha Upanishad: “*Of a certainty the man who can see all creatures in himself and himself in all creatures, knows no sorrow (7).*”

The Advaita Vedanta philosophical underpinning proved useful in framing many of the questions that my laboratory pursued for its first 10 years or so. But slowly, I began to feel that the topics we were addressing were somewhat off the mark, a personal sense of “*hamartia*” (borrowed from theatrical literature, the flaws leading to the downfall of a tragic character). All of our experiments had been done with pure cultures and using one model organism, the venerable *E. coli*. But in such isolation from the rest of the living world, as revealing as it was, the work felt limited. How else might I look and get just a bit closer to understanding what is life?

**Fig 4:**
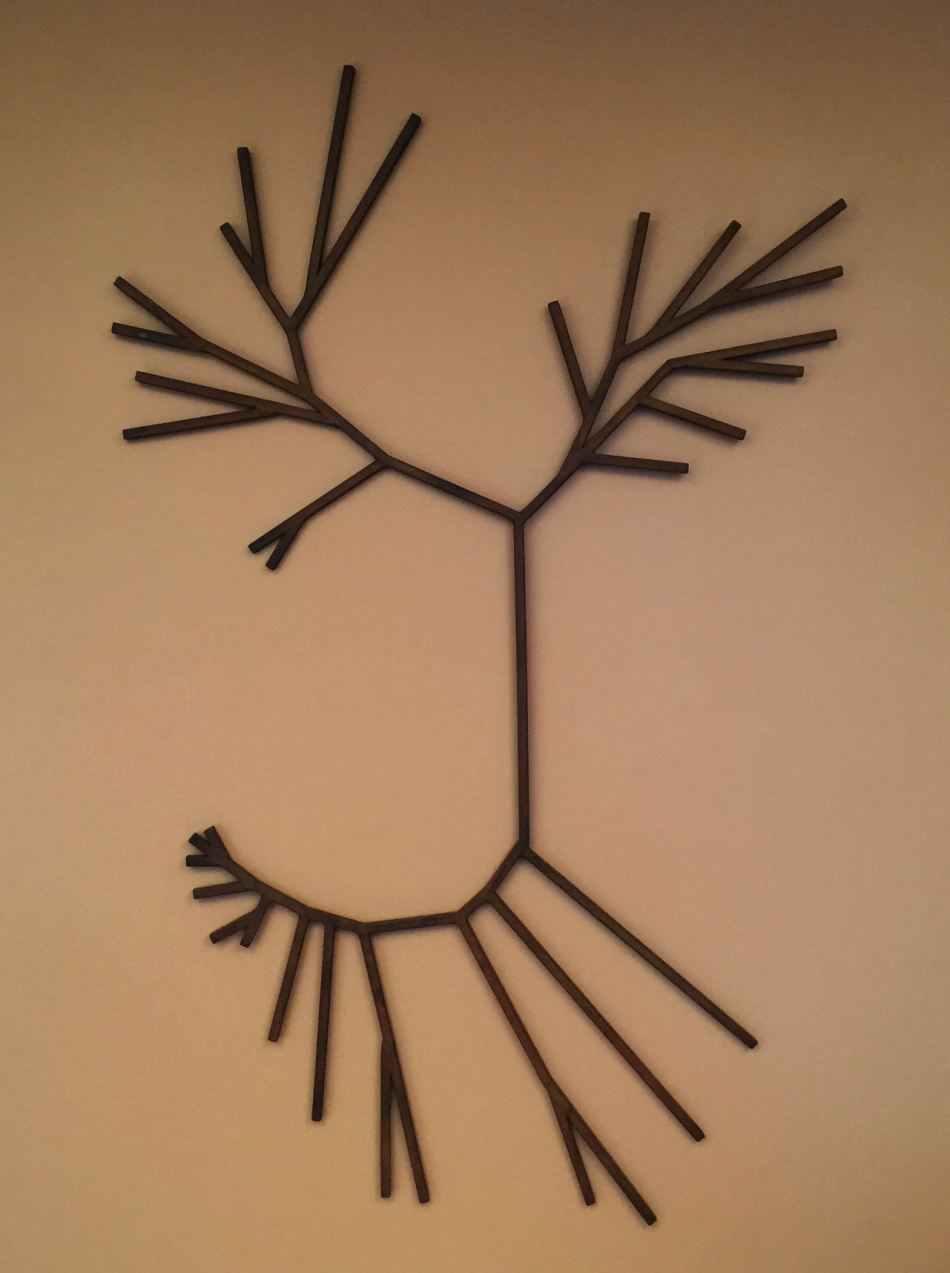
Rusted iron sculpture, representing the three-domain tree of life, that hung behind my office desk.

For me, our approach to asking questions no longer felt entirely right. Something was missing, and I felt I needed a change of mind (*metanoia*). As I searched for new directions, my philosophical outlook also began to change. I no longer felt at ease with “*what is true for E. coli is true for the Elephant.*” At a philosophical level, I felt the need to understand not only their similarities but also their differences. Differences that resulted from their divergent natural histories. I had been so absorbed by molecular biology, I had tended to forget ecology and evolution. I was partially awakened by thoughts emanating from two writings: Theodosius Dobzhansky’s “*Nothing in biology makes sense except in the light of evolution”* (9) and Evelyn Hutchinson’s “*"The ecological theater and the evolutionary play (10).”* The importance and inseparability of ecology and evolution became ever more apparent with Carl Woese’s determination of a universal phylogeny based on sequence alignments (11, 12) and the discovery of previously unimaginable microbial diversity by Vigdis Torsvik (13) and Norm Pace (14) and their colleagues (Fig. 4). These discoveries changed my mind. Henceforth, understanding ecosystem function at the molecular level would be my preferred starting point when asking questions about what life is.

We started by taking baby steps in that direction. Initially, we attempted to develop reproducible systems in which to analyze nonlethal interactions involving two or more species on solid surfaces. But with the realization that we did not even understand how a single species behaved on a surface, we quickly focused on studying many different species individually and how they adapted to growth on surfaces. Thus, we found ourselves doing genetics on biofilms (15). Once we began to make inroads in those inquiries, we dared move into multispecies systems (16). Those studies eventually led to us to study the chemical exchanges between species (17). And along the way, we dared explore diverse natural settings (18).

Was there a different philosophy underlying the shift in mindset, which led us to ask such different questions? Yes. I felt the need to go beyond understanding *“the motion of the atoms*” (replication, transcription, and translation, i.e., molecular biology). The new mindset was about understanding the interrelatedness and interactions that result from the undeniable fact that the drama of the evolutionary play is taking place in the ecological theater. From my perspective, a little-known text points in the direction of the philosophical underpinnings of the question of ecosystem function. It is an essay by Julian Davies—a long-time friend and personal hero—entitled “*Everything depends on everything else* (19).*”* This is how Julian explains the title in his opening sentences:

“The title of this article is a translation of a credo of the Haida people, an important Northwest Indian band who live on the Queen Charlotte Islands off the coast of British Columbia. It refers to the interdependent and interactive relationship between people, animals, and their natural environment, a concept that governs their lifestyle.”

He uses this Haida credo as his philosophical foundation from which “*to discuss the nature of the interconnectivity between and within microbial cells, their hosts, and the environment, and the fundamental role that bioactive small molecules play in the kingdom of microbes.”* I find that quite inspiring and very much in keeping with how my trajectory has evolved as I have meandered from Vedic to Buddhist thought. The universal entanglement or interconnectedness expressed in the Haida credo is also wonderfully summarized, not so much in the concept of Atman = Brahman but in the age-old tenet of Buddhist philosophy:

This is, because that is.

This is not, because that is not.

This is born, because that is born.

This dies, because that dies (20).

All of life is interconnected. Can we ever reach a molecular understanding of the interconnectedness that brings about ecosystem function? I do not know, but I can offer some direction. Here is one last philosophical thought, this one coming from the recent past and close to home. It is from Henry David Thoreau, a key member of New England’s transcendentalist movement. In his classic essay “Walden,” he exhorts us to “*simplify, simplify”* (21). Yes, ecosystems are almost invariably extremely complex. But to understand them, they can be (and perhaps even should be) simplified. I think our lab laid a grain of sand on this approach in some of the last questions we addressed experimentally, for example, when we studied the interactions between the alga *Emiliania huxleyi* and the marine bacterium *Phaeobacter inhibens* (22) or when we generated and studied a simplified, representative bacterial community of maize (23). In performing these studies, we were but one among many who sought to gain a better understanding of the interconnectedness of ecosystems by simplifying them. Only time will tell how our efforts helped guide others along the way, if at all. Yet the path was splendid.

In the meantime, I remain in awe of what is life.
